# A methodology for fast assessments to the electrical activity of barrel fields *in vivo*: from population inputs to single unit outputs

**DOI:** 10.3389/fncir.2014.00004

**Published:** 2014-02-05

**Authors:** Jorge J. Riera, Takakuni Goto, Ryuta Kawashima

**Affiliations:** ^1^Department of Biomedical Engineering, Florida International UniversityMiami, FL, USA; ^2^Department of Functional Brain Imaging, Institute of Development, Aging and Cancer, Tohoku UniversitySendai, Japan

**Keywords:** CSD, LFPs, brain current sources, neuronal activity, cerebral cortex, barrel field

## Abstract

Here we propose a methodology to analyze volumetric electrical activity of neuronal masses in the somatosensory barrel field of Wistar rats. The key elements of the proposed methodology are a three-dimensional microelectrode array, which was customized by our group to observe extracellular recordings from an extended area of the barrel field, and a novel method for the current source density analysis. By means of this methodology, we were able to localize single barrels from their event-related responses to single whisker deflection. It was also possible to assess the spatiotemporal dynamics of neuronal aggregates in several barrels at the same time with the resolution of single neurons. We used simulations to study the robustness of our methodology to unavoidable physiological noise and electrode configuration. We compared the accuracy to reconstruct neocortical current sources with that obtained with a previous method. This constitutes a type of electrophysiological microscopy with high spatial and temporal resolution, which could change the way we analyze the activity of cortical neurons in the future.

## Introduction

Currently many efforts are focused on decrypting canonical working principles of cortical microcircuits in mammalians. To this end, the barrel cortex of rats has been a very useful animal model. *In vivo* extracellular electric recording from these barrels provides information about the activity of large populations of neurons with an excellent temporal resolution. Although the extracellular electric recording technique was launched in the middle of the 19th century, it is now recapitulating its role with the rapid development of silicon-based microelectrode arrays (MEA). With the technological advances in the micro-electromechanic systems (e.g., deposition, lithography, etching, die-preparation, Wise, 2005), MEAs with high spatial resolution are gradually being built with a variety of not only microelectrode local configurations (e.g., tetrodes, octodes, polytrodes) but also shank spatial arrangements (e.g., linear or “laminar,” planar and three-dimensional) (Ulbert et al., 2001; Csicsvari et al., 2003; Buzsáki, 2004; Blanche et al., 2005; Kipke et al., 2008; Du et al., 2009; Ogawa et al., 2011; Riera et al., 2012). MEAs with three-dimensional formats are ideal to obtain volumetric recordings from multiple barrels, a crucial step to understand trans-laminar and tangential interactions in the cortical microcircuits with an acceptable spatial and temporal resolution (Riera et al., 2012).

Unfortunately, the extracellular electric potentials do not represent directly the ionic flows generated by excitable membranes in active neuronal ensembles, i.e., the volumetric density of current sources *C*(*t*), but instead they are far-field external reflections of these electric currents through a highly conductive extracellular medium. Accurate biophysical models that included realistic profiles of the electric conductivity are required to properly characterize these external reflections at each particular cortical region. In order to have a good estimation of the current source density (CSD) *C*(*t*) inside a cortical region, extracellular electric potentials need to be observed, usually with respect to a common reference, from a large number of microelectrodes homogeneously distributed inside that region. This is named the CSD analysis. A priori information about the brain current sources is always required to uniquely solve the inverse problem underlying any CSD analysis. Evidence that brain current sources are actually smooth over extended regions within the barrel cortex has been accumulating over the last decade. Despite its clear value, this constraint has not been explicitly introduced in previous methods for CSD analysis.

In this study, we propose a new methodology for performing CSD analysis on volumetric extracellular recordings from the barrel cortex of Wistar rats that is based on:

- the framework of generalized smoothing splines to introduce spatial a priori constraints on the CSD **C**(*t*) (i.e., the vCSD method).
- a volume conductor model that includes realistic observations of the conductivity profile for the barrel cortex of Wistar rats (Goto et al., 2010).
- a three-dimensional silicon-based probe (NeuroNexus Technologies, Inc., http://www.neuronexustech.com/) customized in particular for the barrel cortex of this type of rats.

We apply the proposed method to assess specific features of the current sources in the barrel cortex of adult Wistar rats undergoing whisker deflections. First, we determine the spatial extent of early thalamic inputs into layer 4 of the cortex and use it as a gold-standard to evaluate the performance of our method. In addition to obtaining volumetric current source patterns associated with local field potentials (LFP) during single whisker stimulation, we concurrently determine the spatiotemporal profiles of single cortical neurons by combining our method with those used for spike detection and single-unit classification (Quiroga et al., 2004; Sakata and Harris, 2009) from multiunit activity (MUA). Also, we use simulations to evaluate the stability of our method for different noise levels and electrode grid resolutions. For illustration purposes, we compare the performance of our method with that resulting from the use of an alternative method previously proposed in the literature to perform CSD analysis with three-dimensional MEAs (i.e., the iCSD3D method, Łẹski et al., 2007). MATLAB scripts for the iCSD3D method are available at http://www.neuroinf.pl/Members/szleski/icsd.html.

## Materials and methods

### Animal preparation

All experiments were performed following the policies established by the Animal Care Committee at Tohoku University (Sendai, Japan). Adult Wistar rats (7–11 weeks of age, male) were used in the experiments. Animals were first anesthetized with intraperitoneal (IP) injections of urethane (1.2 g/kg), and immobilized with a stereotaxic system (Narishige, Japan) that comprises ear bars and a mouth/nose clamp. If necessary, an extra dose of urethane was administrated. Before surgery, all whiskers were trimmed to 1 cm. The right somatosensory barrel cortex was exposed through a craniotomy (5 mm in diameter, centered 2.4–2.5 mm posterior to the Bregma and 5.8–6.0 mm lateral from midline) and a small patch of dura matter was carefully removed. Non-conductive paraffin oil (Nacalai tesque) was applied over the exposed brain tissue to keep the cerebral cortex moistened. Two other craniotomies with 1 mm in diameter were made at the left posterior and right posterior parts to the lambdoidal suture to set the ground and reference screws, respectively. These screws were attached to the skull by dental cement and in direct contact with the brain's surface.

### A three-dimensional silicon-based MEA

**Figure 1:**
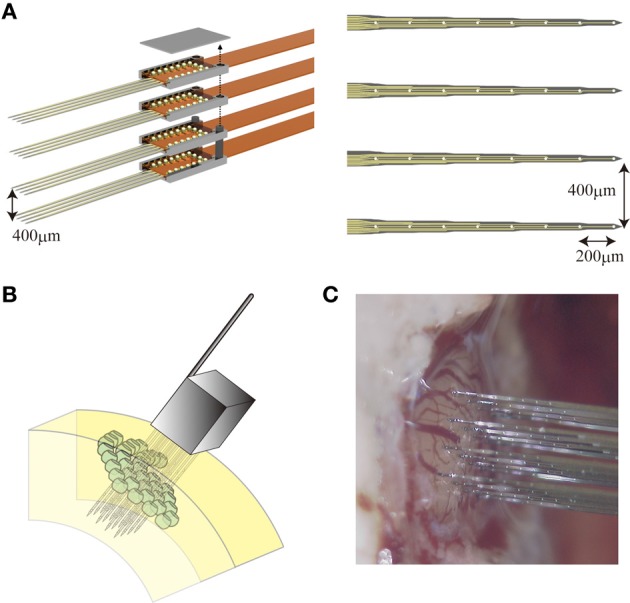
**The 3D array**. (**A**, left) Micro assembly of four planar probes a courtesy of Neuronexus Tech. (**A**, right) Each planar probe comprises 32 microelectrodes with 400 μm inter-shank distance and 200 μm inter-electrode distance along each shank. An illustration **(B)** and a photograph **(C)** of the 3D array right before its implantation into the somatosensory cortex of a rat.

In this study, we used a three-dimensional silicon-based microelectrode array (3D array) that was customized in collaboration with NeuroNexus Technologies, Inc. The 3D array is composed of multiple 2D planar probes (4 shanks each with 8 microelectrodes, 200 μm inter-electrode distance) which are bound together (400 μm inter-shank distance) using micro assembly technique (Figure 1A). Figure 1B shows an illustration of the 3D array after being inserted into a virtual barrel field of a rat. A picture with the probe in position to be inserted into an actual somatosensory barrel cortex is shown in Figure 1C. Each 3D probe has 128 microelectrodes in total covering a volumetric region of interest (ROI) of about 2 mm^3^, which means 4–9 adjacent barrels. By means of this 3D array, changes in the distribution of the extracellular electric potentials in such a ROI are observed with high temporal resolution.

### Three-dimensional recordings of extracellular electric potentials

We implanted the customized 3D array into the exposed somatosensory barrel cortex in a way that the tip of each shank was at a depth of 1600 μm. Due to the strong reactivity of the brain tissue compared to other probe formats (e.g., laminar and planar), the insertion of the 3D array constitutes one of the most difficult steps of the proposed methodology. Repelling forces make the tissue easily bent and recover upon attempted insertion of the probe. Therefore, we applied a gradual insertion method where the 3D array is iteratively inserted two steps forward (200 μm) and one step backward (100 μm) until a designated depth is reached. Each insertion was observed using a customized rotating digital microscope (KH-1300, Hirox; Narishige).The probe insertions were performed with a micromanipulator (Combi 25Z; Luigs and Neumann Feinmechanik, Ratingen, Germany) and the procedures were always monitored on the digital display of the micromanipulator's control system (SM5; Luigs and Neumann). Each shank of the 3D array was carefully painted with a lipophilic neuronal tracer carbocyanine (DiI, D282; Invitrogen) to reveal its actual position from histological images, which were obtained after each recording section. Experiments were early terminated for those rats with considerably cortical bleeding due to perforations of pial vessels. We also excluded from the analysis several rats whose histological images show signs of cortical swelling or abnormal lamination. It took us some years to master this insertion protocol.” In most of the cases, deformations of the cortical tissues were observed neither during the experiment nor on the postmortem images.

For comparison with the iCSD3D method (Łẹski et al., 2007), half of microelectrodes were excluded, resulting in an array of 64 microelectrodes, to mimic a 3D array with equidistant microelectrode arrangement (400 μm inter-electrode distance and 400 μm inter-shank distance). The 3D array was connected to the main amplifiers (PZ-2, Tucker-Davis Technologies, TDT) through a couple of 64 channel ZIF-Clip® headstages (ZC64; TDT). The PZ-2 amplifiers were connected to a signal processor unit (RZ-2; TDT) by optical fibers. The electric potentials at the microelectrodes were recorded with respect to the reference electrode, and with a sampling frequency of 25 kHz.

Individual whiskers were deflected by the piezoelectric bimorph actuator (TAYCA, Japan). The deflection angle, frequency and interval for each whisker deflection were set to 7.2°, 1 Hz, and 100 ms, respectively. To that end, square pulses with these parameters were programmed in MATLAB and the resulting signals were used to energize a piezoelectric bimorph actuator through the D/A converter (PCI-6259, National instruments, USA) and the piezo driver (PCD-001, General Photonics, USA). For each condition, we recorded 100 trials.

### Histology

After recordings, rats were perfused with 4% paraformaldehyde in 0.1 M sodium phosphate buffer saline solution, and their postmortem brains were kept in the same solution overnight. After that, the fixed brains were cut tangentially to the brain surface in 100 μm thickness by a tissue sectioning equipment (Vibratome 1000-plus; Leica Microsystem). To reveal the barrels, the sections were treated with 3,3′-diaminobenzidine (DAB, Sigma D8001) and cytochrome C oxidase from horse heart (Sigma, C2506) following the protocol by Civillico and Contreras (2006). Co-localized immunostaining images that reveal the shank positions and barrels were obtained by using an upright fluorescent microscope (SZX16, Olympus).

### Data preprocessing

**Figure 2:**
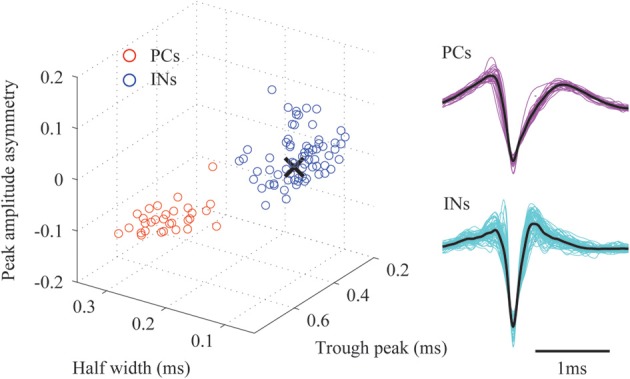
**A classification of the detected spikes**. The **right panel** shows the classified mean spike waveforms of the excitatory pyramidal cells (PCs) and interneurons (INs). Black lines denote their mean spike waveforms. **Left panel** shows the spike waveforms as projected onto the three-dimensional feature space. The black cross indicates the mean spike waveform of the detected spiny stellate (SS) cells.

The extracellular electric potential comprises two types of electrophysiological signals (Gray et al., 1995), i.e., the LFPs, which reflect spatiotemporal superposition of synaptic inputs to the neuronal populations, and the unit activity, which captures the action potentials produced by neurons in close proximity to the microelectrodes. To obtain LFPs from the raw data, we applied a Butterworth band-pass filter with cut-off frequency of 1 Hz and 500 Hz. Event-related potentials (ERPs, Φ (*t*), *t* = −50 − 100 ms) evoked by whisker deflections were calculated by averaging LFPs over 100 trials. Another band-pass filter with cut-off frequency of 500 Hz and 8 kHz was applied to the raw data. From the resulting high frequency components, we extracted MUA by negative edge detection *with a* threshold of 4 times the standard deviation and 1.5 ms dead time. Twenty samples (i.e., eight and twelve samples prior and posterior to the spike troughs, respectively) of the detected spikes were used for classification. Spikes at each microelectrode were divided into putative excitatory pyramidal cells (PCs) and interneurons (INs) by two-step clustering strategy (Ogawa et al., 2011). First, we represented the spikes using four-level Haar wavelets. From the resulting 20 wavelet coefficients, 10 representative coefficients were selected as the input for cluster analysis using the Kolmogorov–Smirnov test. The cluster analysis was performed using the superparamagnetic clustering method (Blatt et al., 1996) followed by a manual clustering strategy to avoid obvious outliers and misclassifications. The aforementioned data processing was carried out using the free-downloaded MATLAB toolbox, “Wave Clus” (Quiroga et al., 2004). Second, we extracted three features from the mean waveform of each classified spike cluster, i.e., the peak amplitude asymmetry, half width and trough peak. We applied k-means clustering method to these features and we finally obtained two spike clusters (Figure 2). Based on the three features, we assumed that spikes whose waveforms show “wide” and “narrow” shapes were generated by putative PCs and INs, respectively (Sakata and Harris, 2009). The separability of these clusters was tested by the Hotelling's T-squared test (*P* = 0.022). It is well known that spiny stellate (SS) cells in Layer 4 are one of the INs in the neocortex. The spike's duration for SS cells is around 0.6 ms, which is within the range of that for the INs (i.e., 0.27–0.65 ms) but different from that for the PCs, i.e., from 0.70 to 1.50 ms (Tierney et al., 2004). Therefore, based only on its duration it is difficult to distinguish a spike fired by a SS cell from one fired by a GABAergic INs. Meanwhile, a study using intracellular recording showed that SS cells in the stimulated barrel respond around 6–8 ms after the deflection (Armstrong-James et al., 1992). Based on this criterion, we selected the microelectrodes located around layer 4 of the barrel corresponding to the stimulated whisker. We picked up IN-like spikes observed at these microelectrodes in the post-stimulus period from 6 to 8 ms, and defined them as putative SS cells. The spiking times of PCs, INs and SS cells at each microelectrode were used as triggers to compute the spike-triggered average of the electric potentials (STAPs). The black cross in Figure 2 corresponds to the features extracted for the SS cells. Clearly it is hard to distinguish SS cells from GABAergic INs in terms of spiking characteristics.

### The vCSD method

**Figure 3:**
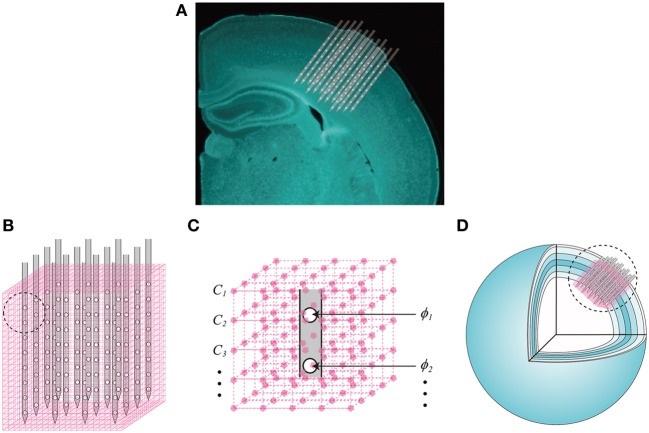
**Definition of the current source grid and the volume conductor model. (A)** A composed fluorescent image of a Nissl stained coronal section of the rat brain and the illustration of the 3D array inserted into the somatosensory barrel cortex. **(B)** The current source grid (magenta) is defined for a ROI covered by the inserted 3D array. A magnified picture of the current source grid corresponding to the black-dashed-circle is also shown **(C)**. Each grid point has a vCSD value *C*_*j*_,(*j* = 1, 2, …, *M*) and the electric potential observed at the *i*th microelectrode is denoted by ϕ_*i*_, (*i* = 1, 2, …, *N*). **(D)** The spherical inhomogeneous and anisotropic *(SphIh)* volume conductor model. Six concentric spherical shells represent the layers of the somatosensory cortex, and each shell has particular radial and tangential conductivity values (Goto et al., 2010).

Neither the LFPs nor the unit activity independently represent the ionic flows across cell membranes, i.e., the volumetric CSD. Instead, they are external reflections of these electric currents through a highly conductive extracellular medium. The key component of our proposal is the vCSD method to reconstruct these trans-membrane ionic flows for both types of extracellular electric potentials. The main idea underlying the vCSD method is illustrated in Figure 3. Consider a 3D array of *N* = *n*_*x*_ × *n*_*y*_ × *n*_*z*_ microelectrodes implanted in a neocortical ROI. The position of the probe inside the cortical regions is determined from the DiI traces left in the histological sections (Figure 3A). The symbols, *n*_*x*_ and *n*_*y*_ denote the numbers of shanks in the *x* and *y* directions, respectively, and *n*_*z*_ represents the number of microelectrodes on each shank. The actual positions of these microelectrodes are ${\vec{r}}_{e}^{i}\in R^{3}$, (*i* = 1, 2, …, *N*) and the electric potentials observed at these microelectrodes are denoted by ϕ_*i*_. The ROI is divided into *M* = *m*_*x*_ × *m*_*y*_ × *m*_*z*_ cubic microscopic volumes. We called the resulting cubic mesh, with inter-node distance *d*, as the *“current source grid”* (Figures 3B,C). Discrete point current sources *I*_*j*_ (j = 1, 2, …, *M*) are defined at the grid points ${\vec{r}}_{s}^{j}\in R^{3}$ of the current source grid. Note that the relationship between the actual vCSD value *C*_*j*_ and *I*_*j*_ at each grid point is represented by *C*_*j*_ = *I*_*j*_/*d*^3^.

Under the validity of the quasi-static approach for the propagation of the electric field inside the brain tissue (Plonsey and Heppner, 1967), the Poisson equation is useful to relate the electric potentials and the current sources inside the brain

(1) $$\nabla \;\cdot \;\left(\overset{↔}{\sigma }\nabla \phi \right)=-C,$$

where $\overset{↔}{\sigma }$ denotes the conductivity tensor. After solving the above partial differential equation independently for each time instant, the current sources defined on the discrete grid and the resulting electric potential ϕ_*i*_ at the *i*th microelectrode can be related by the following biophysical model, known as the forward problem (Goto et al., 2010)

(2) $$\begin{array}{l} \phi_{i}=G({\vec{r}}_{e}^{i},\;{\vec{r}}_{s}^{j},\;\Theta )C_{j}\\ \;=G_{ij}C_{j}, \end{array}$$

where *G* is the generalized Green's function that is determined by the ROI's geometry, the boundary conditions, and the conductivity profile of the brain tissues, i.e., the volume conductor model. These physical properties are summarized in the parameter set Θ in function *G*. Note that we are using Einstein symbolic sum notation. As a consequence of the superposition of the electric fields, the resulting electric potential at each microelectrode reflects contributions from all current sources. The relationship between electric potentials at all microelectrodes in the 3D array and the current sources at the grid points can be represented by the following algebraic equation

(3) Φ=GC

where Φ = [ϕ_1_ ϕ_2_ … ϕ_*N*_]^*T*^ and *C* = [*C*_1_ *C*_2_ … *C*_*M*_]^*T*^ are vectors, and **G** is the discrete generalized Green's function matrix

(4) $$G=\left[\begin{array}{cccc} G_{11} & G_{12} & \cdots & G_{1M}\\ G_{21} & G_{22} & \cdots & G_{2M}\\ \vdots & \vdots & \ddots & \vdots \\ G_{N1} & G_{N2} & \cdots & G_{NM} \end{array}\right].$$

For simplicity, we have ignored the time dependency in our definitions. The vCSD method consists of estimating **C** from measurements of Φ, which represents in fact an ill-posed inverse problem.

### The volume conductor model

The use of a realistic volume conductor model **G** constitutes one of the most significant differences between the vCSD method and other conventional methods for CSD analysis (e.g., Łẹski et al., 2007; Potworowski et al., 2011). In some theoretical studies, inhomogeneity and/or anisotropy in the electric conductivity have been considered (Holt, 1998; Pettersen et al., 2006) on the basis of experimental evidence, e.g., in the cerebellum (Nicholson and Freeman, 1975; Okada et al., 1994) and in the neocortex (Hoeltzell and Dykes, 1979). However, most of CSD methods in the literature assumed an infinite, homogeneous and isotropic volume conductor model, denoted in this paper by the Green's function (*InfH*, **G**_inf_). It was demonstrated in the past that changes in the electric conductivity do not significantly affect the results obtained with the classic CSD method, which is based on the second-order spatial derivative of the electric potentials (Mitzdorf and Singer, 1980). Conversely, Goto et al. (2010) showed that misspecification of the volume conductor model in terms of both geometry and conductivity profile affect dramatically a more contemporary method, i.e., the iCSD3D method (Łẹski et al., 2007). Goto et al. (2010) proved that the somatosensory cortex of rats can be locally approximated by six spherical shells, which were easily determined from fluorescent images of brain sections labeled by the fluorescent Nissl staining. Also, detailed measurements of the electric conductivity profile in this particular cortical region, revealed the existence of significant anisotropies (Goto et al., 2010). Based on this previous study, we used a spherical inhomogeneous and anisotropic (*SphIh*) volume conductor model, with corresponding Green's function **G**_sph_, for the somatosensory cortex of rats (Figure 3D), and used the mathematical strategy proposed by De Munck and Peters (1993) to calculate **G**_sph_ numerically.

### Suppressing the effect of noise on the CSD reconstruction

Commonly, the number of point current sources is larger than the number of microelectrodes *M* >> *N*, and also, the linear operator based on function $G({\vec{r}}_{e}^{i},\;{\vec{r}}_{s}^{j},\;\Theta )$ has a non-trivial null space; hence, the matrix **G** has an incomplete rank and is poorly conditioned. The use of a priori information about C has become a standard way to deal with this problem, giving rise to the well-known *“distributed inverse solution”* family. The low resolution electrical tomography (LORETA), which results from a vector laplacian penalization to the optimization functional for the primary current density, constitutes, so far, one of the most acknowledged distributed inverse solutions for macroscopic EEG data (Pascual-Marqui et al., 1994). LORETA can be interpreted within the context of the *general smoothing splines* introduced by Wahba (1990) to solve noisy operator equations (Riera et al., 2006). LORETA inverse solution warrants not only smoothness of the reconstructed C but also forces it to be minimal on the boundary of the brain. Technically, the LORETA type of inverse solution of equation (3) results from minimizing the optimization functional *o*(**C**) = ‖Φ − **GC**‖^2^ + λ‖**LC**‖^2^ respect to the CSD vector C. The matrix L is the discrete spatial Laplacian operator defined as

(5) $$\begin{array}{l} \;L=\frac{6}{d^{2}}(W-E)\\ {\left[W\right]}_{ij}=\left\{\begin{array}{cc} \frac{1}{6}, & if\;\left\|{\vec{r}}_{s}^{i}-{\vec{r}}_{s}^{j}\right\|=d\\ 0 & \text{otherwise} \end{array}\right\},\;\forall i,\;j=1\ldots M \end{array}$$

where **E** is the *M* × *M* identity matrix.

Finally, the solution of the weighted linear regression problem is:

(6) $$\hat{C}={\left(G^{\prime }G+\lambda L^{\prime }L\right)}^{-1}G^{\prime }\Phi$$

The estimation of the hyper-parameters λ is a problem of considerable importance, since it tells us about the accuracy of the electrophysiological instrument, the quality of the data in terms of the S/N ratio as well as the degree of smoothness to be introduced for the unknown vector C. In this paper, we used the generalized cross validation (GCV) method to estimate λ (Wahba, 1990). Therefore, the optimal λ minimizes the following evaluation function *E*(λ)

(7) $$E(\lambda )=\frac{{\left\|P\Phi \right\|}^{2}}{{\left[tr(P)\right]}^{2}}$$

where the projecting matrix *P* is defined as

$$P=E-G{\left(G^{\prime }G+\lambda L^{\prime }L\right)}^{-1}G^{\prime }.$$

The vCSD method was applied to grand-average ERPs and STAPs. To that end, immunostaining images were used to define the current source grid relative to the position of the microelectrodes. Based on the imprints of the shanks and insertion depth of the 3D array, we defined a rectangular current source grid, comprising *M* = 30 × 30 × 28 grid points with *d* = 50 μ m inter-grid distance. The CSD **C(*t*)** for each time instant was estimated by solving Equation (6) with **G** = **G**_sph_. The iCSD3D was also applied to the ERPs and the respective CSD in the current source grid was estimated. Note that in order to remove the dynamic effect of the signal observed at the reference electrode, we applied the average reference operator (Pascual-Marqui, 1999) to the Green's function matrices, ERPs and SRPs (Offner, 1950; Bertrand et al., 1985).

### Effect of volume conductor model on the CSD analysis

Goto et al. (2010) have evaluated how misspecifications of the conductivity profile and the cortical geometry affect the CSD reconstruction using the iCSD3D method. Such a method is based on the assumptions of an infinite ROI with homogeneous and isotropic conductivity. Goto et al. (2010) found distortions in the CSD reconstructions, especially in the case of CSD distributions with charge-unbalances. In this study, we performed a simulation to ensure that such distortions are minimized by the proposed vCSD method when an appropriate volume conductor model is used. As in Goto et al. (2010), we employed a 3D array (*N* = 9 × 9 × 15 microelectrodes array, 100 μm inter-electrode distance along the shank, 100 μm inter-shank distances). We defined the rectangular current source grid which has *M* = 16 × 16 × 28 grid points with a resolution *d* = 50 μm. Two types of CSDs were simulated. The first type was a sinusoidal function weighted by a Gaussian term, which represents charge balanced CSDs.

(8) $$C_{j}=\{\begin{array}{ll} \sin\left[\frac{2\pi (z_{j}-z_{0})}{T}\right]\exp\left(-\frac{\sqrt{x_{j}^{2}+y_{j}^{2}}}{2l^{2}}\right) & if\;\left|z_{j}-z_{0}\right|<\frac{T}{2}\\ 0 & \text{otherwise} \end{array}$$

where *C*_*j*_ is the value of CSD at the *j*th grid point located in the tangential coordinates {*x*_*j*_, *y*_*j*_} and the radial depth *z*_*j*_, *l* is the full width at half-maximum (FWHM) in the *xy*-plane. The second type of CSD was a pure Gaussian function, which represents charge-unbalanced CSDs.

(9) $$C_{j}=\exp\left(-\frac{\sqrt{x_{j}^{2}+y_{j}^{2}+{\left(z_{j}-z_{0}\right)}^{2}}}{2l^{2}}\right)$$

where *l* is the FWHM in both the *xy*-plane and *z* direction. From these CSD distributions, we simulated electric potentials at the microelectrodes Φ by using equation (3) with the *SphIh* volume conductor model (i.e., **G**_sph_). After that, we performed both the vCSD analysis (i.e., **G** = **G**_sph_) and the iCSD3D method (i.e., **G** = **G**_inf_) to estimate $\hat{C}$ from the simulated data Φ. Finally, the reconstruction errors (RE) for both methods were evaluated from the estimated CSDs by the following criterion

(10) $$\text{RE}=\sqrt{\sum_{j=1}^{M}{\left(C_{j}-{\hat{C}}_{j}\right)}^{2}}/\sqrt{\sum_{j=1}^{M}{\left(C_{j}\right)}^{2}}$$

### CSD reconstruction from noisy data

To assess the sensitivity of the proposed vCSD method to noise, we conducted another simulation study. We employed a cubic current source mesh which had *M* = 24 × 24 × 24 grid points with an inter-grid distance *d* = 50 μm. The following Gaussian type of CSD distribution was used.

(11) $$C_{j}=\exp\left(-\frac{\left\|\vec{r_{j}}-\vec{r_{0}}\right\|}{2l^{2}}\right)$$

where $\vec{r_{0}}$ is the center of the Gaussian function which for all trials was selected randomly within the ROI. The FWHM *l* was fixed at 400 μm throughout this simulation study. The electric potentials at the microelectrodes Φ were calculated from this CSD distribution by using equation (3) with **G** = **G**_inf_. We computed the potentials Φ_β_ that included an additional noise term

(12) $$\Phi_{\beta }=\Phi +\xi$$

where ξ ∝ **N**(0, σ^2^) is a Gaussian noise with zero mean and variance σ^2^. The variance σ^2^ was determined from the sample variance of Φ

(13) $$\sigma^{2}=\beta \sum_{i=1}^{N}{\left(\phi_{i}-\frac{1}{N}\sum_{i=1}^{N}\phi_{i}\right)}^{2}$$

where the parameter β ∈ [0.01, 0.05, 0.1, 0.5] determined the level of noise.

We estimated the CSD distribution $\hat{C}$ from simulated data Φ_β_ by the vCSD method, i.e., Equation (6) with **G** = **G**_inf_, and the iCSD3D method (**G** = **G**_inf_). The REs were calculated for each value of β. Additionally, we evaluated the impact of the resolution of the microelectrode array on the CSD reconstruction by calculating the respective RE for arrays with 200, 300, 400, and 600 μm inter-electrode distances.

### Localization of the barrels

To evaluate the accuracy of the vCSD and iCSD3D methods, we used estimated CSD distributions to detect the barrels corresponding to the particular deflected whiskers. We manually registered the anatomical barrels covered by the current source grid. First, we picked up one *xy*-plane **U** ⊂ *R*^2^ in the current source grid at the approximated depth of layer 4. This plane (*M*_*L*4_ = 24 × 24 grid points) was superimposed with the immunostaining image and the relative position of each grid point was defined as ${\vec{r}}_{k}\in U,\;\left(k=1,\;2,\;\ldots ,M_{L4}\right)$. Note that grid points outside of the microelectrode grid, i.e., the outermost three grid points in both the *x* and *y* directions and the outermost two grid points in *z* direction in the 30 × 30 × 28 sized current source grid, were used to equivalently introduce a free boundary condition that allow us to accommodate outside current sources Łẹski et al. (2007). CSD values at those grid points were ignored.

Second, we defined a binary value *a*_*k*_ at each grid point in the two-dimensional grid plane ${\vec{r}}_{k}$, resulting in a vector **A** = (*a*_1_, *a*_2_, …, *a*_*M*_*L*4__). Third, we defined a barrel space **B** ⊂ **U** manually from the immunostaining images. And finally, the elements of **A** were set by the following criterion

(14) $$a_{k}=\{\begin{array}{cc} 1 & if\;{\vec{r}}_{k}\in B\\ 0 & \text{otherwise} \end{array}$$

We used the binary vector **A** (i.e., the anatomical barrel) as the Gold Standard for evaluating the accuracy with which barrel were detected by the CSD methods. The following thresholding method was used to detect a barrel:

1. We normalized the CSD distribution $\bar{C}({\vec{r}}_{k})$.
  (15) $${\hat{C}}_{n}({\vec{r}}_{k})=\hat{C}({\vec{r}}_{k})/\underset{{\vec{r}}_{k}\in U}{\max}\left(\hat{C}({\vec{r}}_{k})\right)$$
2. We defined another binary vector **F**(α) = (*f*_1_ (α), *f*_2_(α), …, *f*_*M*_*L*4__(α)) as the functional barrel. A threshold α in the interval [0, 1] was used to define the elements of **F**(α) by the following criterion.
  (16) $$f_{k}(\alpha )=\{\begin{array}{cc} 1 & if\;{\hat{C}}_{n}\left({\vec{r}}_{k}\right)\geq \alpha \\ 0 & \text{otherwise.} \end{array}$$
3. We determined the threshold α^*^ in a way that the functional barrel has same area as that of the corresponding anatomical barrel, i.e., difference of the total summation of the components in the binary vectors **A** and **F**(α) is minimized.
  (17) $$\alpha^{*}=\underset{\alpha \in [0,\;1]}{\arg\min}\left|{\left\|A\right\|}^{2}-{\left\|F(\alpha )\right\|}^{2}\right|$$
4. For evaluating the detection accuracy, i.e., the localization error, we used the normalized distance between the anatomical **A** and functional **F**(α^*^) barrels.
  (18) $$\text{Localization Error}=\frac{{\left\|A-F\left(\alpha^{*}\right)\right\|}^{2}}{{\left\|A\right\|}^{2}}$$

In this formalism, the localization errors corresponding to the best and worst detected barrel are 0.0 and 1.0, respectively. We found no computational problems for all barrels analyzed with this method.

### Statistical analysis

The Kolmogorov–Smirnov test was used to determine the wavelet coefficients that better represent the spikes. The Hotelling's T-squared test was used to evaluate the separability of clusters in the spike's parameter space. Pair-based comparisons were performed using the one-tailed *t*-test and Mann-Whitney *U* test for the REs and *Localization Error*, respectively.

## Results

**Figure 4:**
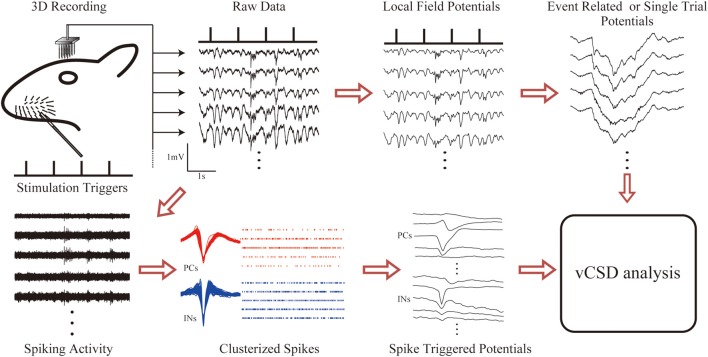
**The methodology for fast assessments to the electrical activity of cortical networks in the barrel field of Wistar rats**. Electric potentials recorded with the 3D array under single whisker deflections are divided into LFPs and unit activities via corresponding band-pass filters (1–500 Hz for LFPs and 0.5–8.0 kHz for unit activity). Event related and single trial potentials are computed from the LFPs. The spike triggered average of the electric potentials (STAPs) for pyramidal cells (PCs) and interneurons (INs) are obtained through spike detection and clustering methods. Finally, the vCSD method is applied to the STAPs as well as to the averaged (ERPs) and single trial LFPs to estimate the spatiotemporal CSD maps associated with single unit activities and population inputs, respectively.

**Figure 5:**
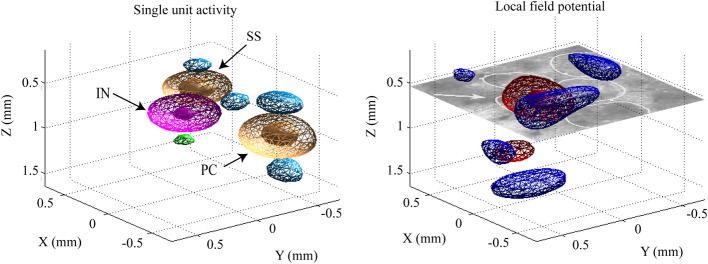
**Example of the CSD distributions estimated for single unit (left panel) and population synaptic (right panel) activities**. The CSD distributions are represented in three dimensional contours. The contours denoted by meshes and patches represent the weak (30% of the maximum) and strong (70% of the maximum) intensity of the CSD, respectively. In the **left panel**, orange and magenta are used for the current sink of the excitatory [pyramidal (PC) and spiny stellate (SS) cells] and inhibitory (IN) neurons, respectively. Blue and green are for the current sources generated by PC/SS and IN, respectively. In the **right panel**, red and blue are used to represent current sink and source, respectively. The CSD maps were estimated from instantaneous ERPs at 8 ms post-stimulus time of a single whisker deflection. On the right frame, the white circles in the histological image denotes the barrels and one of them, indicated by the arrow, corresponds to the barrel associated to the deflected whisker in this particular condition.

**Figure 6:**
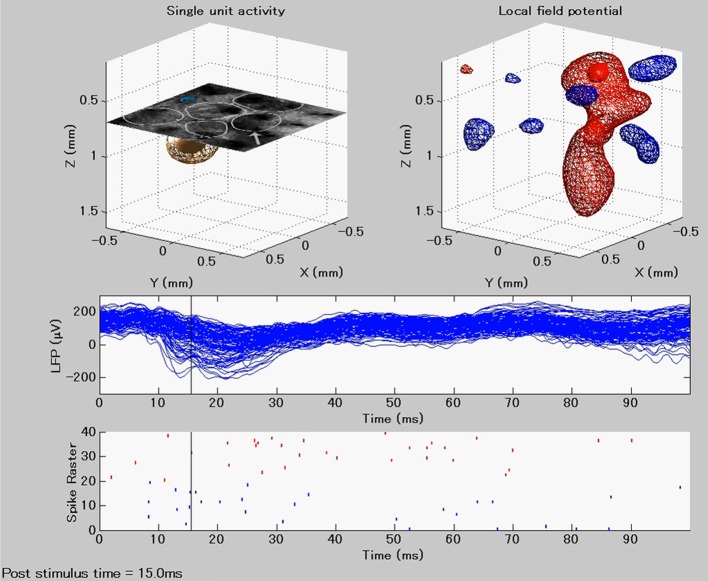
**Instantaneous image taken from the Supplementary Video at time instant (15 ms post-stimulus), indicated by a horizontal black line at the lower panels**. The **upper panels** show single trial spike (left) and LFP-related (right) CSD distributions after a single whisker deflection at time *t* = 0. The **lower panels** show the time courses of the LFPs obtained from each electrode in the 3D array and the respective raster plots of the detected single units.

Figure 4 summarizes the methodology for fast assessments of the electrical activity of cortical networks in the barrel field of Wistar rats. Single whisker evoked-potentials were recorded from the somatosensory barrel cortex by using the 3D array. These potentials were separated into LFPs and unit activities by applying low and high range band-pass filters, respectively. We extracted LFPs for single trial responses, and also computed the ERPs. At the same time, neuronal spikes were detected at each microelectrode, and classified into excitatory PCs and INs. An additional criterion was applied to distinguish SS cells from INs (see Material and Methods). The spiking times of the classified cells were used to compute the STAPs. Cortical current sources associated with single trial LFPs, ERPs, and STAPs were processed through the vCSD method (Figure 5). The example movie of a single trial response can be seen in the Supplementary Video and an explanation for a particular time instant is in Figure 6.

### Effect of volume conductor model on the vCSD analysis

**Figure 7:**
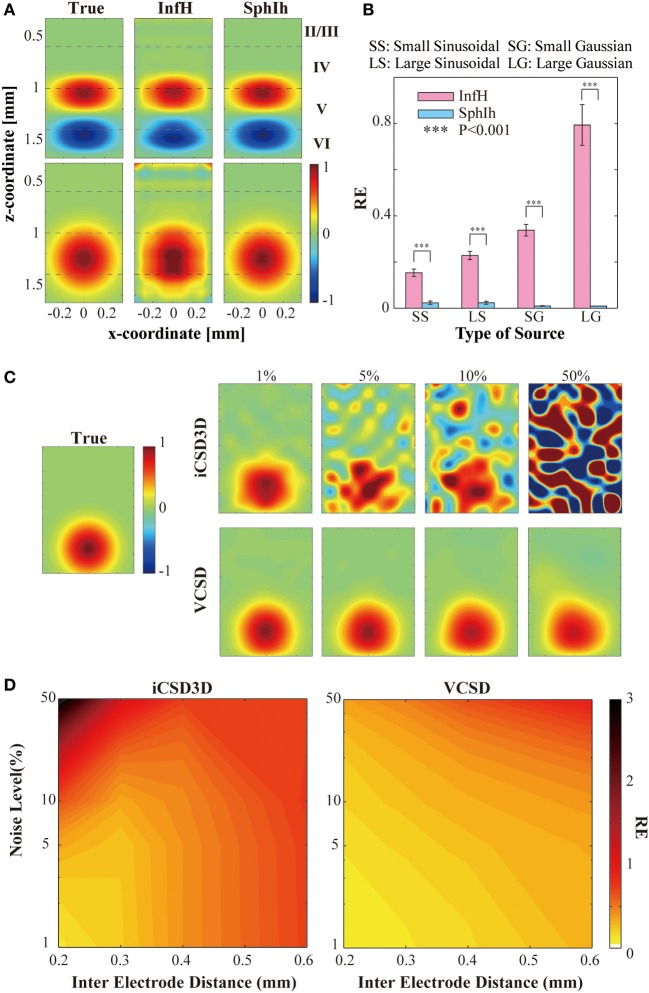
**Results of the simulations studies to evaluate the vCSD method**. **(A)** Example of the CSD reconstructions of large-sized sinusoidal and Gaussian types of CSD of distributions by vCSD method using both InfH and *SphIh* volume conductor models. The dashed lines in each panel indicates the boundaries separating the cortical layers. **(B)** Statistical test to evaluate the reconstruction errors (REs) for *InfH* and *SphIh* volume conductor models. The triple asterisk indicates the threshold of the *p*-value (*P* = 0.001, *n* = 10) used for the one-tailed *t*-test. **(C)** Example of the CSD reconstructions of a Gaussian CSD distribution (panel leftmost) from electric potentials observed by a virtual 3D array which have 200 μm inter-electrode distance. Top and bottom rows are the CSD distributions estimated by the iCSD3D method and the vCSD method with *InfH*, respectively. Each column denotes a different noise level added to the simulated electric potential distributions. **(D)** Contour plots for the reconstruction errors as the functions of the noise level and the inter-electrode distance.

We conducted computer simulations to evaluate the effect on the vCSD method of certain misspecifications in the volume conductor model. Figure 7A shows, for a single trial, the actual current sources (left panels) used to generate the electric potentials, as well as their reconstructions by means of the vCSD method in the cases of employing the *InfH* (center panels) and *SphIH* (right panels) volume conductor models, respectively. The current sources in the upper and lower panels were created using a balanced (*sinusoidal*) and an unbalanced (*Gaussian*) model, respectively. When the *SphI*h volume conductor model was used, the CSD was accurately reconstructed for both small and large sized sources (reconstruction error, REs <2%). However, the CSD reconstructions obtained using the *InfH* volume conductor model showed significant distortions in the spatial configuration with larger REs. These distortions were more prominent for the case of charge-unbalanced models of the current sources. High REs for the charge-unbalanced CSD reconstructed by the iCSD3D method were also reported in Goto et al. (2010). The statistics for the REs reported in Figure 7B were obtained by performing ten single trial simulations with current sources centered at different depths along the cortical lamina.

### CSD reconstruction from noisy data

We have also performed a second simulation study to compare the noise sensitivity and the spatial resolution of the iCSD3D and vCSD methods. To this end, we simulated electric potentials which were contaminated with observational noise at different levels from 1 to 50%. In order to use the original iCSD3D method, we employed in this particular simulation study the *InfH* volume conductor model for both methods. Note that downloadable MATLAB code for the iCSD3D model is only available for *InfH* volume conductor. Additionally, these electric potentials were calculated for silicon-based probes with three dimensional microelectrodes arrays having different spatial resolutions, i.e., from 0.2 to 0.6 mm inter-electrode distances. Figure 7C shows the reconstructed current sources by both iCSD3D (upper panels) and vCSD (lower panels) methods for the particular case of a silicon-based probe with inter-electrode distance of 0.2 mm. In this figure, each column-wise panel shows the CSD reconstruction for different noise levels. Color maps with the REs for both types of CSD analysis methods as a function of the noise level and the inter-electrode distance are shown in Figure 7D. The iCSD3D method was able to correctly reconstruct current sources for low levels of observational noise. However, the REs, in this particular case, increased rapidly with the inter-electrode distance. The situation was dramatically inverted when large noise contamination in the observed electric potentials existed, with a very poor reconstruction for higher resolution MEA. However, substantial improvements were achieved when we reduced the electrode's resolution (i.e., increase inter-electrode distance). These improvements were observed in 50% of noise level only for inter-electrode distances longer than 0.4 mm. Even though they were smaller, significant differences in the REs were found (*P* < 0.001) between the vCSD and iCSD3D methods. This simulation study revealed an intrinsic tradeoff in the iCSD3D method, which results from the lack of a regularization term to stabilize an inverse operator defined from a highly ill-conditioned matrix. Such compromise between the noise level in the data and the spatial resolution of the microelectrode array was not observed in the case of using the vCSD method. The vCSD method kept acceptable performance even at 50% noise level and inter-electrode distance of 0.2 mm. In the current simulation study, we employed 50 trials for each noise level and microelectrode array's resolution.

### Localizing single barrels using the vCSD method

**Figure 8:**
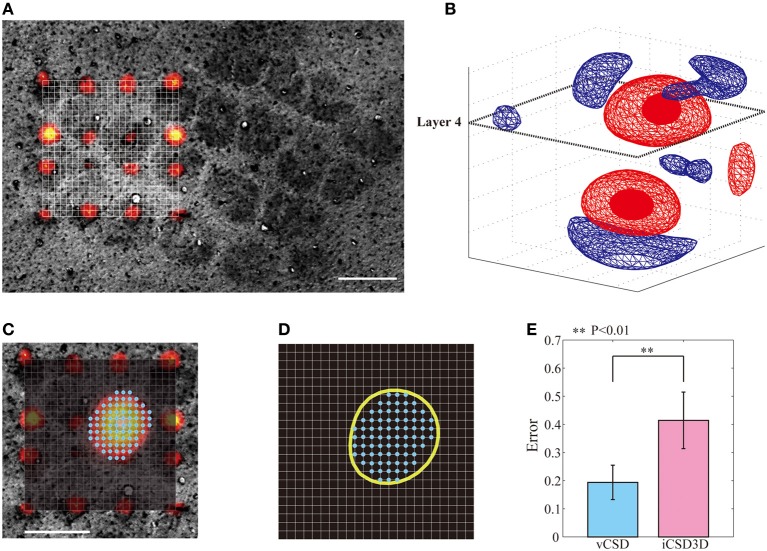
**Localization of barrels by means of the iCSD3D and vCSD methods**. **(A)** CSD distribution at 6–8 ms post-stimulus with an illustration of the *xy*-plane at the level of layer 4. **(B)** Co-registration of the *xy*-plane of the current source grid at the depth of layer 4 and the anatomical image acquired from the cytochrome c oxidase stained tangential brain section of the barrel cortex. The position of the shanks are determined from Dil staining images. **(C)** Superposed pictures in the *xy*-plane of the CSD distribution at the depth of layer 4 and the anatomical barrel denoted by cyan dots. **(D)** The functional barrel (yellow circle) obtained from the *xy*-slice of the CSD distribution and the anatomical barrels. **(E)** Localization errors between the anatomical barrels and the corresponding functional barrels estimated by iCSD3D and vCSD methods. The one-tailed Mann-Whitney *U* Test (*P* = 0.004, *n* = 5) was used to compare the performances of these two methods.

**Figure 9:**
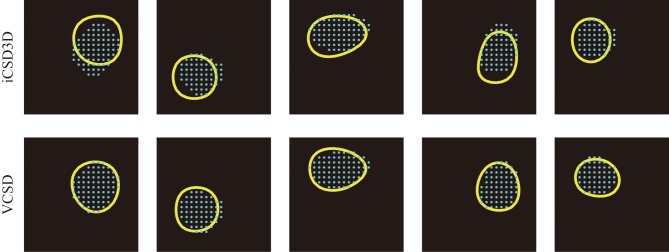
**Five samples of anatomical barrels (cyan dots) and their corresponding functional barrels (yellow circles) estimated by the iCSD3D (top row) and vCSD (bottom row) methods**. It can be seen that the vCSD method provided better estimates of the functional barrels than those obtained by the iCSD3D method. We pointed out that among the main reasons for the inaccuracy of the iCSD3D method are the misspecification of the volume conductor model and/or the effect of systematic noise in the data.

In this study, we used the actual anatomical barrels as the *“Gold standard”* to validate our methodology for single whisker deflection. The main reasons for using the anatomical barrels come from the structure of the barrels and their spatiotemporal synaptic responses to single whisker stimulation. First, the main inputs from the thalamus to the somatosensory barrel field arrive at layer 4 of the cortex, where the SS cells process them. The arriving times of these first sensory inputs are around 6–8 ms after the whisker deflection (Armstrong-James et al., 1992; Wilent and Contreras, 2004). After the SS cells receive these inputs, they increase their activity by self-feedback mechanisms within the corresponding barrel (Feldmeyer et al., 1999). During this period, synapses of the SS cells play the main role in producing post-synaptic potentials. The dendrites of SS cells located inside a particular barrel extend mainly to the center of the barrel, indicating most of these synapses are delimited to the single barrel (Woolsey et al., 1975; Petersen and Sakmann, 2000; Egger et al., 2008). Based on these facts, the synaptic activities of SS cells in response to the single whisker deflections are limited within the corresponding barrel, i.e., current sinks in the period of 6–8 ms after a single whisker deflection are confined within the barrel. This hypothesis has been supported by previous *in vitro* and *in vivo* studies. For instances, *in vitro* field EPSP recordings in the barrel field after the electric stimulation of its center showed that excitatory neuronal circuits within layer 4 are functionally confined to each barrel (Petersen and Sakmann, 2000). Additionally, *in vivo* VSDI imaging, and *in vivo* extracellular recordings by horizontal planar Utah intra-cortical microelectrode array (in combination with spike histogram analysis) showed that the barrels corresponding to the deflected whiskers could be well-localized (Petersen and Diamond, 2000; Petersen et al., 2003). Taking into account the anatomical and functional characteristics of the barrels, we used actual whisker ERPs recorded by our 3D array at this particular time instant to evaluate the performance of the vCSD method. By means of Dil staining, we were able to co-register the CSD distribution and the anatomical barrels, which were clearly determined from the *xy*-images of cytochrome C oxidase immunostaining at the level of layer 4 (Figure 8A). We estimated the CSD distributions in the period of 6–8 ms post-stimulus (Figure 8B). We defined the functional barrels from the CSDs based on a thresholding method. The thresholds were chosen in a way that the functional barrels have the same area as the corresponding anatomical barrels (Figures 8C,D). Both anatomical and functional barrels were represented by binary vectors whose lengths represented the total number of grid points on the *xy*-plane of the current source grid at the level of layer 4. Figure 8E shows localization errors of the functional barrels for reconstruction with the vCSD and iCSD3D methods. The results from single trial comparison are shown in Figure 9. We found that the localization error of the vCSD method was lower than 20% (19.0 ± 6.1%) and this method always produces more accurate reconstructions than the iCSD3D method (41.4 ± 10.1% localization error).

## Discussion

We have demonstrated that, by the combination of mathematical methods and high technology, it is possible to image the activity of neuronal networks from extracellular electric recordings at resolutions unprecedented for electrophysiological methods. For the first time, to our knowledge, images with high spatial-resolution in both the horizontal plane (i.e., cortical columns) and trans-laminar axis (i.e., cortical layers) are obtained from electrophysiological recordings in similar fashions to those achieved via multi-photon fluorescent microscopes. From actual electrophysiological recordings, we reconstructed the CSD distributions at any depth of the barrel cortex separating cortical inputs from their outputs. Additionally, we were able to discriminate spike-related CSD distributions for different types of cells. Our methodology will be quite useful for a variety of applications in neuroscience, from both a biophysical and an electrophysiological point of view. For example, from the cable theory for PCs, it is known that the spatial summation of the trans-membrane current must be zero. However, by means of the vCSD analysis, it was possible to evaluate this hypothesis for the case of STAPs (Riera et al., 2012). Also, in this latter study, the same analysis was applied to evaluate multipolar contributions to the LFP recordings. These issues are important in the light of recent interests in elucidating fundamental principles of the EEG and MEG genesis. Also, by the proposed methodology, it will be possible to identify layers, and determine detailed interactions between these layers but also columns (barrels) in behaving rats. Our group is currently using the proposed methodology to determine the spatial codifiers of the whisker velocity and direction (*unpublished data*). Our methodology could be extended in the future to study other cortical regions and species.

Methods to perform CSD analysis on data recorded with three-dimensional MEAs are still under development, with only different volumetric version of the inverse CSD (iCSD, Pettersen et al., 2006) method available in the literature (i.e., the iCSD3D method, Łẹski et al., 2007; the kCSD method, Potworowski et al., 2012). The main idea behind these methods is to use interpolating splines to represent the extracellular electric potentials, and thus to indirectly introduce specific priors for the density of current sources *C*. The iCSD3D method was recently improved by formulating it in the context of reproducing kernel Hilbert spaces and introducing a Tikhonov regularization strategy (Potworowski et al., 2012). These authors used a cross-validation technique to determine the best value for the regularization parameter λ whenever the data are corrupted with noise. In contrast, the proposed method is the first introducing smoothing constraints directly to the brain current sources *C* over extended regions of the barrel cortex to solve the inverse problem underlying the CSD analysis. The performance of the proposed method was evaluated, and compared with that for the iCSD3D method, using simulated data with different noise levels and electrode grid resolutions.

Although the iCSD3D method can be trivially generalized to more complex volume conductor models, it originally assumed for simplification that the brain tissues are homogeneous and isotropic. In this study, we claim that more realistic volume conductor models for the brain tissues of interest must be used to considerably improve the accuracy of the three-dimensional CSD analysis. The iCSD3D method has been applied in the past to averaged extracellular electric potentials obtained from the deep forebrain of one adult male Wistar rat during whisker stimulation, with an insertion/recording strategy that allow to cover a volume of (2.8 × 3.5 × 4.9) mm with a total of 140 electrodes. However, we have evaluated its performance in this study using not only simulations but also an experimental paradigm for a gold standard.

The method developed in this work is directly applicable to perform CSD analysis whenever the following conditions are met: (a**)** recordings of extracellular potentials are performed with a tridimensional MEA, (b**)** the conductivity profile of the area of study is layer-wise inhomogeneous and anisotropic, and (c**)** the geometry is approximately spherical. As a consequence of its extensive use by the community, we have developed the entire methodology for the particular case of the barrel cortex of rats. Although the application to other cortical areas of the rats might be straightforward, its use in other species and brain regions must be carefully evaluated in accordance with the respective conductivity profiles and geometries (e.g., somatosensory cortex of cats, Hoeltzell and Dykes, 1979; CA1 of guinea pigs, Holsheimer, 1987; cerebellum of turtles, Okada et al., 1994; visual cortex of monkeys, Logothetis et al., 2007).

## Future developments

The application of the multi-photon fluorescent imaging technique to study the brain constitutes one of the most remarkable achievements in the era of the colored revolution in neuroscience (Denk et al., 1990; Vonesch et al., 2006). By combining this technique with the bulk-loading method for membrane-permeable Ca^2+^-indicator dyes (Stosiek et al., 2003), both sensory-evoked and ongoing activity in neuronal populations have been observed *in vivo* from rodent/cat neocortex with the spatial resolution of single neurons. Recently, the technique has benefited from the latest technological and methodological advances in the evaluation of both neuronal spiking (Wallace et al., 2008) and volumetric activity (Göbel et al., 2007; Cheng et al., 2011). However, there are several limitations of the multi-photon fluorescent imaging technique, which make the methodology proposed in this study a better option for observing neuronal population activities in a variety of neuroscience problems. First, except when using voltage-sensitive fluorescent dyes, multi-photon microscopic imaging commonly constitutes an indirect measurement of the actual membrane potentials (i.e., it senses slow changes in the intracellular Ca^2+^ concentrations). As a consequence, it is hard to distinguish subthreshold neuronal activity (i.e., post-synaptic inputs) from spiking (i.e., axonal outputs). In comparison to Ca^2+^-indicator dyes, the sensitivity of voltage-sensitive fluorescent dyes for imaging subthreshold electrical activity is excellent. Unfortunately, the latter lack single-cell spatial resolution *in vivo* (Kuhn et al., 2008) and are deficient in terms of the S/N ratio. Also, alterations in the cellular physiology have been associated with the use of voltage-sensitive fluorescent dyes (Mennerick et al., 2010). Second, multi-photon imaging still suffers from poor time resolution even though a lot of technical progresses have been made recently (Göbel et al., 2007; Planchon et al., 2011), Thus far, precisions of a few milliseconds have been achieved by combining some of these methods (Grewe et al., 2010), but actual reconstructions of spike dynamics, propagation and timings from fluorescence traces are just about to happen. Third, the neocortical tissues are high light-scattering media, which results in an imaging-depth limit (Theer and Denk, 2006). By combining regenerative amplifiers (Theer et al., 2003) with genetically encoded calcium indicators, even layer 5 (up to 800 μm) have been recently imaged *in vivo* (Mittmann et al., 2011) although image resolution at that depth is poor. In principle, MEA could be combined with silicon photonics to take advantages of optical applications quickly developed in this decade. The methodology proposed in this study could also benefit from recent advances in MEA fabrication. For example, to improve interaction with neural cells, microelectrodes built from nanoscale bioactive coatings (e.g., polymers) have been proposed (Richardson-Burns et al., 2007). By means of multiplexing and telemetry techniques, miniaturized and wireless multi-channel systems are speedily developing for recording neural signals from behaving small animals (e.g., rats, Szuts et al., 2011).

The MATLAB code for the vCSD analysis is available at the following website http://web.eng.fiu.edu/jrieradi/.

### Conflict of interest statement

The authors declare that the research was conducted in the absence of any commercial or financial relationships that could be construed as a potential conflict of interest.
